# Effect of Remineralized Collagen on Dentin Bond Strength through Calcium Phosphate Ion Clusters or Metastable Calcium Phosphate Solution

**DOI:** 10.3390/nano10112203

**Published:** 2020-11-04

**Authors:** Hyeryeong Kim, Aerin Choi, Mi-Kyung Gong, Hae Ryoun Park, Yong-Il Kim

**Affiliations:** 1 Department of Orthodontics, Dental Research Institute, Pusan National University, Yangsan 50612, Korea; dalbit222@naver.com (H.K.); pnudh_aerin@naver.com (A.C.); 2 Department of Oral Pathology, School of dentistry, Pusan National University, Yangsan 50612, Korea; mkgong10@gmail.com; 3 Periodontal Disease Signaling Network Research Center (MRC), School of Dentistry, Pusan National University, Yangsan 50612, Korea; 4 Dental and Life Science Institute, Pusan National University, Yangsan 50612, Korea

**Keywords:** CPICs, metastable calcium phosphate, dentin remineralization, dentin bonding, dentistry, conservative

## Abstract

This study aimed to investigate whether dentin remineralization and micro-tensile bond strength increase when using calcium phosphate ion clusters (CPICs) or metastable Ca-P. After being etched, each dentin specimen was designated into four groups and treated with the appropriate solution for 1 min: 100% ethanol, 2 and 1 mg/mL of CPICs, and metastable Ca-P. The specimens were then prepared for scanning electron microscopy (SEM), transmission electron microscropy (TEM) imaging, a matrix metalloproteinases inhibition assay, and the micro-tensile bond strength test. To compare among the groups, one-way analysis of variance was performed. In the SEM imaging, with a rising concentration of CPICs, the degree of remineralization of dentin increased significantly. The metastable Ca-P treated specimens showed a similar level of remineralization as the 1 mg/mL CPICs treated specimens. The TEM imaging also revealed that dentin remineralization occurs in a CPICs concentration-dependent manner between the demineralized dentin and the resin layer. Furthermore, the results of micro-tensile bond strength showed the same trend as the results confirmed by SEM and TEM. We demonstrated that a 1 min pretreatment of CPICs or metastable Ca-P in etched dentin collagen fibril can achieve biomimetic remineralization and increase micro-tensile bond strength.

## 1. Introduction

The management of demineralized tooth surface is an important issue in dental practice. Treatment of the caries-affected enamel/dentin layer has been approached from two perspectives; biomimetic remineralization and conservative treatments using restorative materials [1,2,3].

In particular, biomimetic remineralization strategies employed in dentistry mimic the progressive dehydration mechanism of natural bio-mineralization or proceed in the absence of apatite seed crystallites in dentinal collagen matrix by non-classical crystallization theory [4]. Among them, Wang J. et al. introduced the remineralization protocol of dentin collagen by a meta-stabilized amorphous calcium phosphate (ACP) solution [5]. They concluded that ACP played a role in bio-mineralization for the demineralized dental tissues. Recently, Shao C. et al. investigated new types of calcium phosphate ion clusters (CPICs), which can establish a biomimetic crystalline-amorphous mineralization frontier to induce the epitaxial growth of enamel [6]. A few nanometer-sized CPICs can form the building blocks of ACP [7]. From these biological strategies, they showed that a bottom-up biomimetic remineralization delivery system could help the mineral-free, resin-infiltrated collagen matrices to be remineralized, and suggested the clinical translation of bio-remineralization approach for remineralizing thick layers of carries affected dentin [8].

However, in conservative dentistry, demineralized or caries-affected enamel/dentin is currently managed by the bonding system. The bonding system creates a unique form of dental tissue engineering known as the hybrid layer, created as the resin monomer penetrates into the demineralized dentin collagen matrix [9]. Unfortunately, hybrid layer is still the weakest link between the adhesive and dentin. The hydrolytic degradation of exposed collagen would undermine the integrity of the hybrid layer, the resin/dentin bonding, and, eventually, the dental restoration [3]. To improve the stability of hybrid layer, clinical studies have been conducted to increase dentin’s bond strength by preventing hydrolytic degradation of the hybrid layer [6,7]. Matrix metalloprotenase (MMP) inhibitors such as chlorhexidine digluconate, 10-methacryloyloxydecyl dihydrogen phosphate (MDP), Cu, and Zn ions were the well-known promising approach [10]. However, the created hybrid layer is still incomplete, which can cause micro-leakage between the dentin and resin, and hence require conservative re-treatment [3].

Therefore, the translational study to improve the structural environment of hybrid layer should be needed through biomimetic remineralization. Several studies have already successfully performed intra- and extra-fibrillar bio-mineralization in dentin collagen [11]; however, few studies have incorporated collagen fibrillar mineralization into dentin bonding. The purpose of this study was to investigate the effect of remineralized collagen in dentin on the dentin bond strength through CPICs or a metastable Ca-P solution. Specially, we aimed to determine what concentrations of CPICs and metastable Ca-P solutions cause the exposed collagen remineralization and whether remineralized collagen increases micro-tensile bond strength.

## 2. Materials and Methods

### 2.1. Preparation of CPICs and Metastable Calcium Phosphate Solution

Unless otherwise specified, the following chemicals were used without further purification: calcium chloride dihydrate (CaCl_2_·2H_2_O; 99.0%, Sigma-Aldrich, St. Louis, MO, USA), triethylamine (TEA, (C_2_H_5_)_3_N; 99.5%, Sigma-Aldrich, St. Louis, MO, USA), phosphoric acid (H_3_PO_4_; 85% in H_2_O solution, Sigma-Aldrich, St. Louis, MO, USA), ethyl alcohol (C_2_H_5_OH; Samchun, Seoul, Korea), hexamethyldisilazane (HMDS; C_6_H_19_NSi_2_; 99.0%, Sigma-Aldrich, St. Louis, MO, USA), glutaraldehyde (C_5_H_8_O_2_; 25% aqueous solution, Sigma-Aldrich, St. Louis, MO, USA), chlorhexidine gluconate solution (C_22_H_30_Cl_2_N_10_; 0.015 mL/mL, BUKWANG PHARM.Co., Seoul, Korea), etchant (35% phosphoric acid solution; Ultra Etch, Ultradent, South Jordan, UT, USA), primer/adhesive (Adper ScotchBond Multi-Purpose Plus, 3 M, Nonrovia, CA, USA), and composite resin (Z350, 3 M, Nonrovia, CA, USA). Deionized water was used in the experiment, and all solutions were filtered through 0.22 µm Millipore films before use. Non-carious human third molars were used for this study. The use of human tooth tissue specimens followed a protocol that was approved by the ethical committee of Pusan National University Dental Hospital (PNUDH-2020-026).

CPICs were synthesized as specified by Shao C. et al. [6]. Two solutions were prepared for the synthesis of CPICs. Solution A, 0.20 g of CaCl_2_·2H_2_O and 3.8 mL of TEA were added into 80 mL of ethanol and then ultrasonicated (BRANSON, Danbury, CT, USA) for five min. Solution B, 70 µL of H_3_PO_4_ was added into 20 mL of ethanol and stirred thoroughly. Solution B was dropped into solution A, with slight agitation, and then CPICs (2 mg/mL) formed in the solution.

The metastable calcium phosphate solution was synthesized as specified by Oyane A. et al. [12]. Briefly, the solution was prepared by dissolving NaCl (142 mM), K_2_HPO_4_·3H_2_O (1.50 mM), and CaCl_2_ (3.75 mM) in ultrapure water and buffering the solution to pH 7.4.

### 2.2. Dentin Collagen Remineralization through CPICs and Metastable Ca-P Solutions

Tooth dentin specimens with a 1 mm thickness were prepared under the dentin–enamel junction, with a low-speed diamond saw (Strauer Accutom-50, Ballerup, Denmark). Each sample was etched with 35% H_3_PO_4_ (Ultra Etch; Ultradent, South Jordan, UT, USA) for 15 s. After etching each sample, washing and air-drying were simultaneously performed for 5 s, to keep the samples slightly wet, to prevent collapse of collagen.

After being etched and washed, each dentin specimen was immediately submerged in a 1.5 mL tube containing one of the four solutions for 1 min: (1) 100% Ethanol (EtOH, control), (2) 2 mg/mL of CPICs solution, (3) 1 mg/mL of CPICs solution, and (4) metastable Ca-P solution. The tooth specimens were then prepared for scanning electron microscopy (SEM, JSM-7900F, JEOL, Peaboy, MA, USA) imaging, without separate washing and drying, following the protocol of Tay et al. [13].

### 2.3. Micro-Tensile Bond Strength (MTBS) Test on Dentin–Resin Bond

To expose the mid-coronal dentin, molars were dissected parallel to the occlusal plane, using a low-speed diamond saw. The exposed surface was then polished with 320, 600-grit silicon carbide discs (SiC), for 60 s, each to remove the smear layer. The tooth blocks were then prepared for resin build-up. The process is as follows: The exposed side of the prepared dentin block was etched with 35% phosphoric acid for 15 s. After etching each sample, washing and air-drying were simultaneously performed for 15 s, to keep the samples slightly wet. After etching and washing, one of the five solutions were applied for 1 min, on the exposed side of the prepared dentin block: (1) 100% EtOH (control), (2) 2 mg/mL of CPICs solution, (3) 1 mg/mL of CPICs solution, and (4) metastable Ca-P solution, or (5) chlorhexidine gluconate solution. The exposed side was blown slightly with air, to remove excess solution, and then primers and adhesives were applied according to manufacturer’s instruction. Subsequently, the composite resin on the corresponding surface was built up 3 to 4 times, to a length of 5 mm. The samples from each group were stored in 37 °C, distilled water (DW), for 24 h. Each sample was then serially cut perpendicular to the bonding interface to obtain dentin–resin beams with precision cutting. Each beam was then fixed to the MTBS tester (BISCO; Schaumburg, IL, USA), using a cyanoacrylate adhesive, and the micro-tensile bond strength was recorded at a crosshead speed of 1 mm/min [14].

### 2.4. Field-Emission Transmission Electron Microscopy (FETEM) Observation of Collagen Remineralization in the Hybrid Layer

For the transmission electron microscopy (TEM) imaging, each dentin–resin beam was fixed in Karnovsky’s fixative, post-fixed in 1% osmium tetroxide, and dehydrated in an ascending ethanol series, following the protocol [13]. The 30–100 nm thick sections were prepared by the focused ion beam (IM-4000, Hitachi, Tokyo, Japan) and examined, without further staining, using FETEM (TALOS F200X; FEI, Hillsboro, OR, USA).

### 2.5. Matrix Metalloproteinases (MMPs) Inhibition Assay

To investigate the inhibitory effect of CPICs and metastable Ca-P solutions on MMPs in dentin, a generic MMP Assay Kit (SensoLyte^®^ Generic MMP Assay kit, AnaSpec Inc., Fremont, CA, USA) was used. To expose the mid-coronal dentin, molars were dissected parallel to the occlusal plane, using a low-speed diamond saw. The exposed surface was then polished with 320, 600-grit SiC, for 60 s, to remove the smear layer. After storing the samples for 24 h in deionized water, at 37 °C, the teeth were cut perpendicular to the exposed surface into a 1.0 mm × 1.0 mm × 4.0 mm beam, to obtain a total of 18 beams. The dentin beams obtained were incubated in 35% phosphoric acid for 15 s and then washed with deionized water for 15 s. Subsequently, the acid-treated dentin beams were randomly divided into six groups (*n* = 3 for each group) and soaked for 1 min in the corresponding solution: (1) 100% EtOH (control), (2) 2 mg/mL of CPICs solution, (3) 1 mg/mL of CPICs solution, (4) metastable Ca-P solution, and (5) chlorhexidine gluconate solution. The other group used an MMP inhibitor as a positive control. Then, the beams were immediately immersed in a 96-well plate containing 250 μL of a generic MMP substrate and incubated at 37 °C for 2 h. The beams were then removed and the total MMP activity was spectrophotometrically determined by measuring the absorbance of each well, at 412 nm, in a plate reader (Synergy HT, BioTek Instruments, Winooski, VT, USA).

### 2.6. Statistical Analysis

To compare among the groups, one-way analysis of variance (ANOVA) with Bonferroni’s multiple comparison tests was performed. The normality of the data was confirmed by Shapiro–Wilk test. All statistical analyses were conducted, using the statistical software (*R language program*, version 3.3.2; R Foundation for Statistical Computing, Vienna, Austria, 2016).

## 3. Results

### 3.1. Dentin Collagen Remineralization through CPICs and Metastable Ca-P Solutions

**Figure 1:**
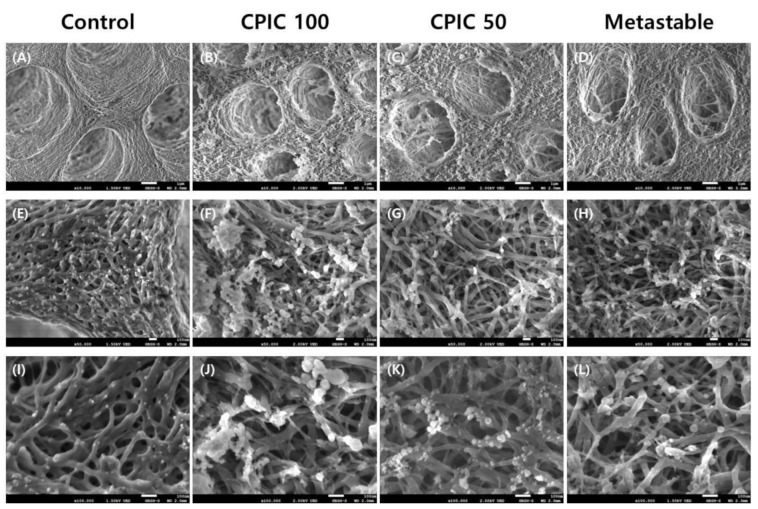
Scanning electron microscopy (SEM) images of remineralized dentin. The first column (panels A, E, and I) is an acid-etched dentin specimen (control); the second and third columns are calcium phosphate ion clusters (CPICs)-treated dentin specimens (CPIC 100 and CPIC 50, respectively); and the fourth column is a metastable Ca-P-solution-treated dentin specimen (simply referred to as metastable). Each horizontal line represents a different magnification (10,000×, 50,000×, and 100,000×, respectively; scale bars on each row represents 1 μm, 100 nm, and 100 nm, respectively). (**A**,**E**,**I**) Control shows the collagen network of the 35% phosphoric acid-etched native dentin surface at the different magnification 10,000×, 50,000×, and 100,000×, respectively; (**B**,**F**,**J**) A bundle of minerals deposited on the surface of the acid-etched dentin surface with the treatment of CPIC 100 at the different magnification 10,000×, 50,000×, and 100,000×, respectively; (**C**,**G**,**K**) Fewer minerals were found on the remineralized dentine surface with the treatment of CPIC 50 at the different magnification 10,000×, 50,000×, and 100,000×, respectively; (**D**,**H**,**L**) A similar amount of remineralization was found on the metastable Ca-P treated dentine surface at the different magnification 10,000×, 50,000×, and 100,000×, respectively. The same results are shown at different magnifications.

SEM and FETEM images were used to determine the ability of the two types of calcium phosphate containing solutions to induce remineralization in dentin. Two concentrations of CPICs (2 and 1 mg/mL, referred to as CPIC 100 and CPIC 50, respectively) and one concentration of metastable Ca-P solution were applied to dentin specimens for 1 min. SEM images of acid-etched dentin revealed the exposed collagen fiber network surrounding the dentinal tubule (Figure 1A). After the 1 min treatment in the calcium phosphate containing CPICs and metastable Ca-P solutions, remineralized dentin was confirmed with the presence of numerous calcium phosphate minerals on the etched dentin surface, compared to the control, and remineralization was found not only on the dentin surface, but also in the intertubular dentin collagen fibrils (Figure 1B–D).

Notably, with the rising concentration of CPICs, the degree of remineralization of dentin increased significantly (Figure 1B,C). The metastable Ca-P-solution-treated dentin specimens showed a similar level of remineralization as the 1 mg/mL CPICs-treated dentin specimens (Figure 1D).

**Figure 2:**
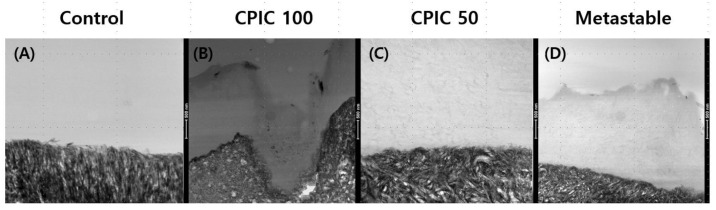
Field-emission transmission electron microscopy (FETEM) images of remineralized dentin. (**A**) An acid-etched dentin specimen (control); (**B**) CPICs 100-treated dentin specimens; (**C**) CPICs 50-treated dentin specimens; (**D**) A metastable Ca-P-solution-treated dentin specimen (simply referred to as metastable). Each image represents the same magnification (see scale bar: 500 nm).

Figure 2 shows that surface remineralization was achieved in demineralized dentin with CPIC 100, CPIC 50, and metastable Ca-P solution pretreated dentin–resin beams, compared to control (Figure 2A). The high-resolution TEM imaging revealed that dentin remineralization occurred in a CPICs-concentration-dependent manner between the demineralized dentin layer and the resin layer (Figure 2B,C). The beams treated with the metastable Ca-P solution also showed a thickened remineralization layer, compared to the control (Figure 2D). The degree of remineralization was lower than that of CPIC 100, but was similar to that of CPIC 50, as shown by the SEM image results (Figure 1C,D).

### 3.2. Micro-Tensile Bond Strength

**Figure 3:**
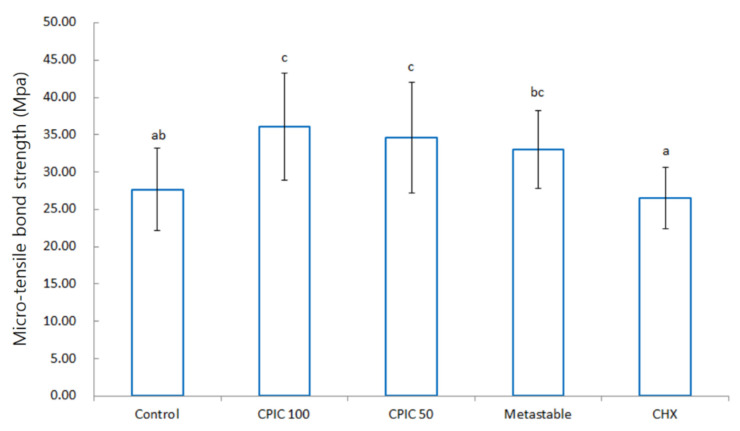
Bar graphs of the micro-tensile bond strengths (MPa) of CPICs, metastable Ca-P solution, and chlorhexidine (CHX) pretreated dentin–resin beams. Each average value is as follows: control, 27.68; CPIC 100, 36.08; CPIC 50, 34.57; metastable, 33.02; CHX, 26.50. The data were analyzed by using analysis of variance (ANOVA) with Bonferroni post hoc test. The same letters (a, b, c) refer to no statistically significant differences.

A MTBS test was performed to determine whether the surface remineralization confirmed by SEM and TEM also affects the actual resin–dentin bond strength. In the MTBS test group, a total of four groups (control, CPIC 100, CPIC 50, and metastable) were tested through SEM, and a chlorhexidine (CHX) group was included as a negative control. A significant difference (*p* < 0.05) was found in the bond strength of resin–dentin bonding between the five groups when the data were examined by using the ANOVA with Bonferroni post hoc test, as shown in Figure 3. The results also showed the same trend as the results confirmed by SEM and TEM. As in the SEM and TEM images, samples treated with CPICs for 1 min were remineralized in a concentration-dependent manner, and the bond strength also increased to an effective value. The sample treated with metastable Ca-P solution for 1 min also showed an increase in bond strength to a level similar to that of CPIC 50. CHX showed a significantly similar or lower value of bond strength compared to the control. From highest to lowest, the MTBS showed increased values in the following order: CPIC 100, CPIC 50, metastable Ca-P solution, control, and CHX.

### 3.3. The Inhibitory Effect of CPICs and Metastable Ca-P Solutions on MMPs in Dentin

**Figure 4:**
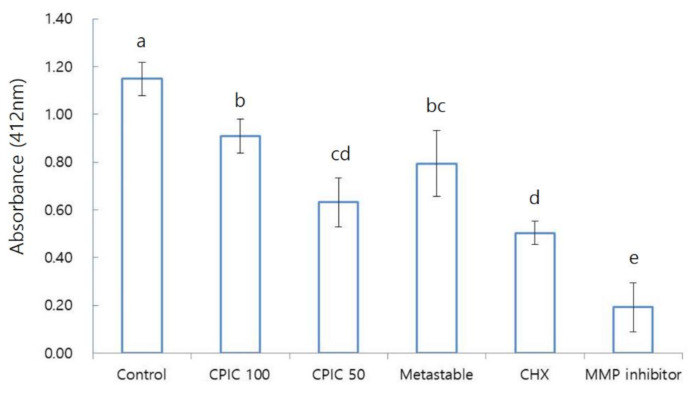
Bar graphs of the matrix metalloproteinases (MMPs) inhibition assay. Each value represents absorbance (412 nm) of CPICs, metastable Ca-P solution, and CHX pretreated dentin–resin beams. MMP inhibitor was used as a positive control. Each average value is as follows: control, 1.148; CPIC 100, 0.910; CPIC 50, 0.632; metastable, 0.794; CHX, 0.503; MMP inhibitor, 0.192. The data were analyzed by using an ANOVA with Bonferroni post hoc test (*p* < 0.05). The same letters (a, b, c, d, e) refer to no statistically significant differences.

We conducted an MMP inhibition assay, with the expectation that the increase in bonding strength by CPICs and metastable Ca-P would be achieved through MMP inhibition. In the MMP inhibition assay, a total of five groups (control, CPIC 100, CPIC 50, metastable, and CHX) were tested through MTBS, and an MMP inhibitor was included as a positive control. There were no significant differences (*p* > 0.05) found in MMP inhibition between the five groups when the data were examined by using an ANOVA with Bonferroni post hoc test, as shown in Figure 4. The results did not show the same trend as the results confirmed by SEM, TEM, and the MTBS study. CPIC 50 showed a significantly higher level of MMP inhibition compared to CPIC 100. MMP inhibition was highest to lowest in the following order: MMP inhibitor, CHX, CPIC 50, metastable Ca-P solution, and finally CPIC 100. Although the trends observed in SEM and TEM images were not followed, clear differences were found between the control and the experimental groups (CPICs or metastable Ca-P).

## 4. Discussion

There are many techniques for restoring damaged teeth. In order to bond the restorative materials to the normal tooth structure, the sealing and adhesion ability of dental adhesives has been steadily developed. Nevertheless, the interface of dentin/adhesive is still the weakest part. The biodegradation of dentin–resin interface causes micro-leakage into the oral cavity overtime after the initial adhesion, eventually leading to restoration failure [15,16]. Interestingly, many studies have reported that the adhesion effect of the dental adhesive immediately after and in the short-term is good, but the long-term retention and stability of the resin adhesion interface to dentin is still questionable [9,16,17].

Vulnerable sites for dentin–resin interface are associated with hybrid layers [9,18]. The formation of a hybrid layer plays an important role in the composite restoration. Looking at the adhesion process, first, a collagen-rich mesh structure is formed on the dentin surface, demineralized to a certain depth by an acidic material. After that, the resin adhesive penetrates into the collagen-rich mesh structure by the primer or the hydrophilic monomer of the dental adhesive. Then, the adhesive is polymerized to form a hybrid layer containing collagen fibers, resin, residual moisture, and hydroxyapatite crystallites [9,18].

However, over time, hydrolysis occurs in the hybrid layer, resulting in a decrease in long-term stability of the resin–dentin bond [9,19]. Various factors are involved in the decomposition of the hybrid layer, but two of these are the hydrolysis of the polymer and exposed collagen fibers [9,19,20,21,22]. It has been reported that collagen fibers exposed in the hybrid layer are hydrolyzed by MMPs, thereby reducing the adhesion of the resin restoration to dentin [15,21,22,23,24]. Therefore, to prevent micro-leakage in the resin–dentin interface, methods for modifying the adhesive, to improve the longevity of the hybrid layer, should be further studied.

From this point of view, the translational research of bio-remineralization approach for remineralizing thick layers of carries affected dentin should be needed. This bottom-up biomimetic approach using calcium phosphate nanoprecursors might play the important role in translational research. This preclinical information can represent an important support to the choice of clinical method [11,25]. This study attempted to remineralize the demineralized collagen matrix in the hybrid layer, improve the structural environment of the hybrid layer, and enhance the adhesion of the resin restoration to dentin through the biomimetic remineralization. Several studies have already successfully remineralized collagen fibers in dentin [2,5], but few studies have incorporated remineralized collagen fiber to the resin-to-dental adhesion. In this study, we investigated the effect of collagen remineralized from dentin through CPICs or a metastable Ca-P solution on dentin’s bonding strength. The remarkable result in our study is the fact that collagen remineralization and MTBS increase by CPICs and metastable Ca-P solutions have the same tendency. The experimental results showed that the tendency found from SEM, TEM, and the MTBS to dentin remineralization was the same. The above results suggest that the increase in dentin adhesion is due to dentin remineralization by CPICs and the metastable Ca-P solution.

The mechanism by which CPICs increase dentin adhesion is related to the concept of ethanol wet bonding and the bio-mineralization of the collagen matrix in dentin [18,26,27]. The pretreatment of a collagen substrate saturated with water with 100% alcohol, instead of water, can provide a more effective opportunity to load hydrophobic monomers into the demineralized collagen matrix, without causing additional substrate shrinkage. That is, it can be made more hydrophobic by applying CPICs, an ethanol-based solution, to the demineralized collagen matrix. In contrast, the demineralized collagen matrix can be made hydrophobic, while the hydrophobic resin mixture is dissolved in ethanol, to make it more hydrophilic. This sequential step facilitates the penetration of the ethanol-solvated hydrophobic resin mixture into the ethanol-saturated collagen matrix. Sadek F.T. et al.’s study showed that the resin-to-dentin bond created by hydrophobic adhesive and ethanol wet bonding did not degrade after 18 months of water storage in the absence of MMP inhibitors [26]. The fact that, in the absence of water, MMP cannot hydrolyze collagen is a major implication for our experiments with CPICs. As described above, ethanol wet-bonding technology greatly contributes to improving adhesion, but due to difficulties in clinical application, an alternative method using a hydrophilic adhesive is currently used. However, our experiments suggest that the difficulty of clinical application of ethanol wet bonding can be overcome by using CPICs.

Biomimetic remineralization of human dentine is a treatment strategy that mimics what occurs in nature, using nanotechnology principles [28]. This strategy replaces water in areas where resin has not penetrated the collagen matrix of the hybrid layer with apatite crystals. Through this, the exogenous collagen-degrading enzyme is excluded, and the endogenous collagen-degrading enzyme is fossilized to increase the life of the resin-to-dentin bond [13,29]. During bio-mineralization, apatite seed crystallites are not present in the organic scaffold. Because of this, biomimetic remineralization of resin–dentin binding uses two polyanionic analogs of the acidic matrix protein. It adopts a bottom-up bio-mineralization approach that individually mimics the sequestration and template function motifs present in naturally occurring matrix protein molecules [18]. This study showed that calcium-phosphate-based analogs, such as metastable Ca-P solution, can induce nucleation and growth of apatite as a collagen-binding template [30,31]. This suggests that the metastable Ca-P solution could be a candidate for improving the durability of resin-to-dentin binding through complete replacement of water in the collagen water compartment and inactivation or silencing of the collagen-degrading enzymes [18].

The differences of adhesion in the beams pretreated with CPICs and metastable Ca-P solution shown in the results suggest that they utilize different dentin-bonding mechanisms. Our results emphasize that both their ethanol wet-bonding strategy and bio-mimetic remineralization strategies can contribute to increasing the bond strength.

In addition, we performed an MMP inhibition assay, as it was expected that the increase in binding strength by CPICs and metastable Ca-P solution would be achieved through MMP enzyme inhibition. However, the experimental results were different than expected (Figure 4). We found that CPIC 50 and metastable had higher MMP inhibition than CPIC 100.

**Figure 5:**
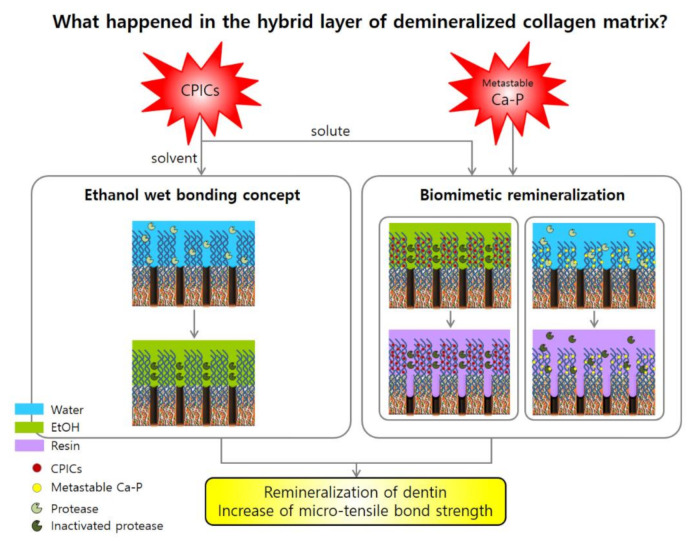
Schematic representation of ethanol-based wet bonding and biomimetic remineralization induced by calcium phosphate ion clusters (CPICs) and metastable calcium phosphate (Ca-P) solution. The CPICs solution increases the bonding power with two mechanisms: The solvent ethanol is the main role of the wet-bonding mechanism. The solute of CPICs increases the micro-tensile bond strength with the same principle as metastable Ca-P, that is, biomimetic remineralization mechanism.

A study by Wang J. et al. showed the process of remineralization of dentin by ACP over a long period, i.e., 30 days [5]. In this regard, our experimental results showed a weaker degree of remineralization than that of the study by Wang J. et al. (Figure 2). The main reason for the difference in the results of the two experiments is, firstly, the short pretreatment time of the solution used for remineralization in our experiment. Kim et al. reported that the biomimetic mineralization process is slow, as it involves at least two kinetically driven pathways and usually takes three-to-four months to complete [4,29]. The second difference is that Wang et al. observed remineralization after partial demineralization of teeth and ACP treatment. In contrast, in our study, we observed remineralization after partial demineralization, CPICs/metastable Ca-P treatment, and resin build-up. Resin build-up may have physically and chemically affected tooth remineralization. Nevertheless, in our study, treatment of CPICs and metastable Ca-P for 1 min resulted in the formation of ion-cluster to the extent that darkness was observed when compared to the control group. This was observed in the TEM image (Figure 2), and it was observed that this clearly affects the bonding force (Figure 3). Moreover, the mechanism for this is shown schematically in Figure 5.

Unfortunately, this study has some limitations. First of all, the sample size was small, and, as explained earlier, the application time for the remineralization process was short. Furthermore, the experimental results of MMP inhibition did not follow the tendency observed in SEM and TEM images. However, if only the control group, CPICs, and metastable Ca-P solution treatment group were compared, there is clearly a difference. Since MMP inhibition is related to long-term bonding, a further long-term experiment is required. Nevertheless, our experiment suggests the possibility of further study and provided interesting evidence for clinical application. The present report tested calcium phosphate solution. However, in the future, it would be interesting to test other recently introduced remineralizing solutions, such as biomimetic nano-hydroxyapatite or bioactive glass [32,33]. On the other hand, dentin and dental adhesives release various molecules with significant potential for application in the repair of damaged odontoblasts and stem-cell-based regeneration following dental caries and operative procedures [34,35]. These bioactive components from dental adhesives can also act as anti-inflammatory, antibacterial, and immunomodulatory elements in the local environment. They can enhance the performance of applications for dental adhesives. Therefore, further studies considering their biological effects on the oral environment should also be needed [36,37].

## 5. Conclusions

This study demonstrated that the one-minute pretreatment of etched dentin collagen fibril with CPICs and metastable Ca-P solutions could achieve biomimetic remineralization and increase micro-tensile bond strength (MTBS). The remineralized dentin surface through CPICs and metastable Ca-P solutions could be seen by FESEM and FETEM images. These interesting results were acquired from ethanol wet-bonding mechanism and biomimetic mineralization strategy. This suggests a new way to overcome the limitations of the hybrid layer that collapses over time and causes micro-leakage. Furthermore, CPICs and metastable Ca-P solutions have the potential to be utilized for sufficient restorative materials in terms of clinical application.
